# Cell Density-Dependent Fibroblast Growth Factor-2 Signaling Regulates Syndecan-4 Expression in Cultured Vascular Endothelial Cells

**DOI:** 10.3390/ijms21103698

**Published:** 2020-05-24

**Authors:** Takato Hara, Shiori Yabushita, Chika Yamamoto, Toshiyuki Kaji

**Affiliations:** 1 Department of Environmental Health, Faculty of Pharmaceutical Sciences, Toho University, Funabashi 274-8510, Japan; takato.hara@phar.toho-u.ac.jp (T.H.); yamamoto@phar.toho-u.ac.jp (C.Y.); 2 Department of Environmental Health, Faculty of Pharmaceutical Sciences, Tokyo University of Science, Noda 278-8510, Japan; bio-organometallics@kf7.so-net.ne.jp

**Keywords:** endothelial cell, FGF-2, proteoglycan, syndecan-4, cell density

## Abstract

Syndecan-4 is a member of the syndecan family of transmembrane heparan sulfate proteoglycans, and is involved in cell protection, proliferation, and the blood coagulation-fibrinolytic system in vascular endothelial cells. Heparan sulfate chains enable fibroblast growth factor-2 (FGF-2) to form a complex with its receptor and to transduce the cell growth signal. In the present study, bovine aortic endothelial cells were cultured, and the intracellular signal pathways that mediate the regulation of syndecan-4 expression in dense and sparse cultures by FGF-2 were analyzed. We demonstrated the cell density-dependent differential regulation of syndecan-4 expression. Specifically, we found that FGF-2 upregulated the synthesis of syndecan-4 in vascular endothelial cells via the MEK1/2-ERK1/2 pathway in dense cell cultures, with only a transcriptional induction of syndecan-4 at a low cell density via the Akt pathway. This study highlights a critical mechanism underlying the regulation of endothelial cell functions by proteoglycans.

## 1. Introduction

Vascular endothelial cells form a monolayer in the lumen of blood vessels and have direct contact with blood. These cells regulate vascular functions, such as the blood coagulation-fibrinolytic system [1], permeability [2], vascular tone [3], and lipid metabolism [4,5]. Several chronic cardiovascular diseases, including atherosclerosis, have been shown to be initiated by injuries in the endothelium. Under physiological conditions, injured vascular endothelial cells release their accumulated fibroblast growth factor-2 (FGF-2). The released FGF-2 then mediates the repair of damaged sites by effectuating cell migration and proliferation [6,7]. Conversely, transforming growth factor-β (TGF-β) is released from α-granules of platelets aggregated at the damaged site [8] to inhibit endothelial cell proliferation [9]. FGF-2 and TGF-β exhibit opposing effects on the regulation of endothelial fibrinolysis [10], as well as proliferation, suggesting that these cytokines confer a coordinated regulation of vascular endothelial cell functions.

Proteoglycans are macromolecules composed of a core protein, with one or more glycosaminoglycan side chains covalently bound to it. The types of proteoglycans expressed in vascular endothelial cells are heparan sulfate proteoglycans and dermatan sulfate proteoglycans. The major products of the former type are the large proteoglycan perlecan [11], found in the basement membrane; the small transmembrane proteoglycan syndecan family, especially syndecan-1 and syndecan-4 [12]; and the small cell-associated proteoglycan, from the glypican family [13]. The small cell-associated proteoglycan includes a small leucine-rich dermatan sulfate proteoglycan, known as biglycan [14]. Proteoglycans synthesized by vascular endothelial cells influence various arterial properties by binding to growth factors, cytokines, receptors, and lipids to modulate their activity [15,16]. In addition, the expressions of these proteoglycans in the vascular wall are dynamically altered during the progression of atherosclerosis [17]. Previously, we have shown that TGF-β_1_ regulates perlecan and biglycan synthesis in a cell density-dependent manner in vascular endothelial cells [18]. Moreover, at a low cell density, the connective tissue growth factor suppresses the synthesis of biglycan, but induces the synthesis of decorin [19]. We hypothesized that FGF-2 also regulates endothelial proteoglycan synthesis in a cell density-dependent manner. With this premise, we aimed to discern the possible mechanism of regulation of endothelial proteoglycan synthesis by FGF-2 and the intracellular signaling pathways involved in the differential regulation.

## 2. Results

### 2.1. Syndecan-4 Expression is Induced by FGF-2 in Dense and Sparse Cultures of Vascular Endothelial Cells

**Figure 1:**
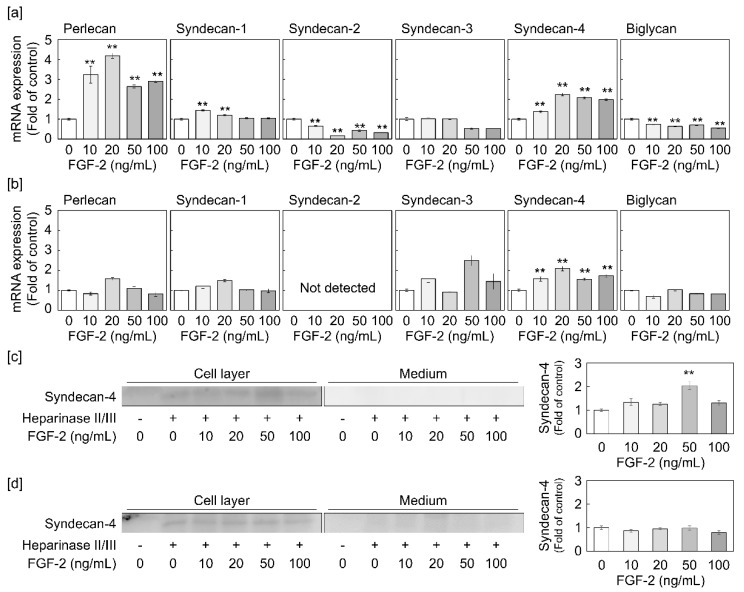
Dose-dependent effects of fibroblast growth factor-2 (FGF-2) on proteoglycan expression in dense and sparse cultures of vascular endothelial cells. The cells were incubated with or without FGF-2 at 10, 20, 50, and 100 ng/mL at 37 °C for 8 h. The mRNA level of the indicated proteoglycans in dense (**a**) and sparse (**b**) cultures of vascular endothelial cells was assessed by qRT-PCR. Values represent the mean ± S.E. of four technical replicates. ** *p* < 0.01, significantly different from the corresponding control (0 ng/mL of FGF-2). The syndecan-4 core protein expression in the vascular endothelial cell layer and conditioned medium from dense (**c**) and sparse (**d**) cultures of vascular endothelial cells was analyzed by western blotting. The bar graphs show the intensity of syndecan-4 in the cell layer in the group treated with heparinase II/III. The values in the bar graphs indicate the means ± S.E. of three samples of the experiments. ** Significantly different from the control, *p* < 0.01.

**Figure 2:**
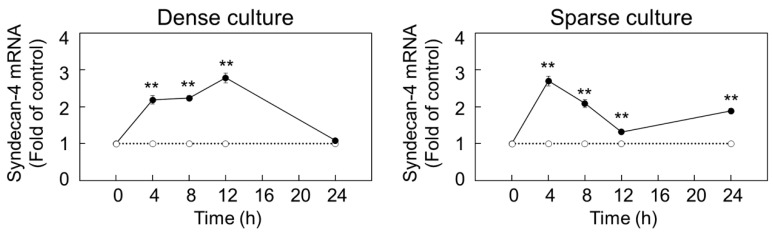
Time-dependent effects of FGF-2 on syndecan-4 mRNA expression in vascular endothelial cells. Dense and sparse cultures (left and right panels, respectively) of vascular endothelial cells were treated with (filled circle) or without (open circle) 20 ng/mL FGF-2 at 37 °C for 4, 8, 12, and 24 h and assessed for the transcript level of syndecan-4 by qRT-PCR. Values represent the mean ± S.E. of four technical replicates. ** *p* < 0.01, significantly different from the corresponding control.

Firstly, we investigated the ability of FGF-2 to regulate the expression of proteoglycans in dense and sparse cultures of vascular endothelial cells. It was observed that perlecan and syndecan-4 transcripts are induced by FGF-2 treatment for 8 h in a dose-dependent manner, while biglycan and syndecan-2 mRNAs were found to be suppressed by FGF-2 in the dense culture (Figure 1a). Furthermore, in the sparse culture, FGF-2 significantly increased syndecan-4 mRNA expression in a dose-dependent manner (Figure 1b). To corroborate these observations, we examined the core protein levels of perlecan and syndecan-4 in the dense culture and those of syndecan-4 in the sparse culture, after treatment with FGF-2. We found an elevated expression of the syndecan-4 core protein in the cell layer from the dense culture treated with FGF-2 at concentrations of 10 ng/mL and higher (Figure 1c). However, FGF-2 did not induce syndecan-4 core protein expression in the cell layer of the sparse culture of vascular endothelial cells (Figure 1d), despite enhancing its mRNA level (Figure 1b). Furthermore, the syndecan-4 core protein did not accumulate in the conditioned medium of either dense or sparse cultures (Figure 1c,d). Thereafter, despite showing an increased mRNA level, an 8 h incubation with FGF-2 failed to induce perlecan core protein expression in both the cell layer and conditioned medium of dense vascular endothelial cells (Figure S1). Taken together, these results indicate that FGF-2 induces syndecan-4 synthesis and perlecan mRNA expression when the cell density is high. Conversely, only syndecan-4 mRNA expression is affected by the growth factor at a low cell density. A time course study indicated that endothelial syndecan-4 mRNA expression could be elevated by FGF-2 at 20 ng/mL after 3 h and longer, in both dense and sparse cultures, with a peak at 12 and 4 h in dense and sparse cells, respectively (Figure 2).

### 2.2. FGF-2 Activates ERK1/2 and Akt in Dense and Sparse Cultures of Vascular Endothelial Cells

**Figure 3:**
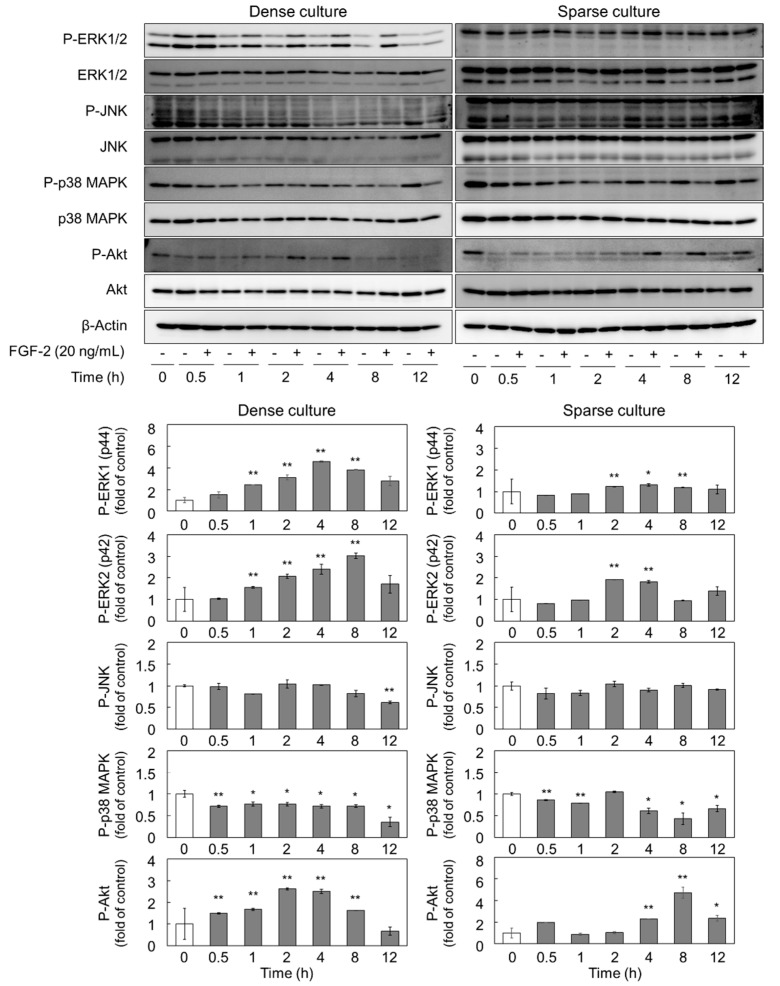
Effects of FGF-2 on the activation of ERK1/2, JNK, p38 MAPK, and Akt in dense and sparse cultures of vascular endothelial cells. Dense and sparse cultures of vascular endothelial cells were treated with or without 20 ng/mL FGF-2 at 37 °C for 0.5, 1, 2, 4, 8, and 12 h. The expression of P-ERK1/2, ERK1/2, P-JNK, JNK, P-p38 MAPK, p38 MAPK, P-Akt, Akt, and β-Actin proteins was assessed by western blotting. The bar graph shows the expression ratio of the phosphorylated MAPKs and phosphorylated Akt in the FGF-2-treated group compared with that in the control group at each time point. The values in the bar graphs indicate the means ± S.E. of three samples of the experiments. Significantly different from the corresponding control, * *p* < 0.05 and ** *p* < 0.01.

With the premise that FGF-2 can activate the mitogen-activated protein kinases (MAPKs, i.e., ERK1/2, JNK, and p38 MAPK) and Akt pathways via the activation of its receptor [20], we investigated the phosphorylation of MAPKs and Akt in dense and sparse cultures of vascular endothelial cells. We found that, in the dense culture, the phosphorylation of ERK1/2 and Akt was increased by 20 ng/mL FGF-2 with 1 to 8 h and 0.5 to 8 h treatment, respectively (Figure 3). Conversely, in the sparse culture, the phosphorylation of ERK1/2 and Akt was elevated by FGF-2 from 2 to 4 h and 4 to 12 h, respectively. Additionally, we observed that the activation of p38 MAPK was suppressed from 1 to 12 h and 4 to 8 h by FGF-2 in dense and sparse cultures, respectively, and the phosphorylation of JNK was unaffected by FGF-2 (Figure 3). The suppression of p38 MAPK by FGF-2 was inconsistent with previous reports showing that FGF-2 activated p38 MAPK, for example, in bovine endometrial cells [21]. As we confirmed the reproducibility of the suppression of p38 MAPK by FGF-2, this phenomenon may be specific for vascular endothelial cells.

### 2.3. FGF-2 Induces Syndecan-4 via the ERK1/2 Pathway in Dense Cultures of Vascular Endothelial Cells

**Figure 4:**
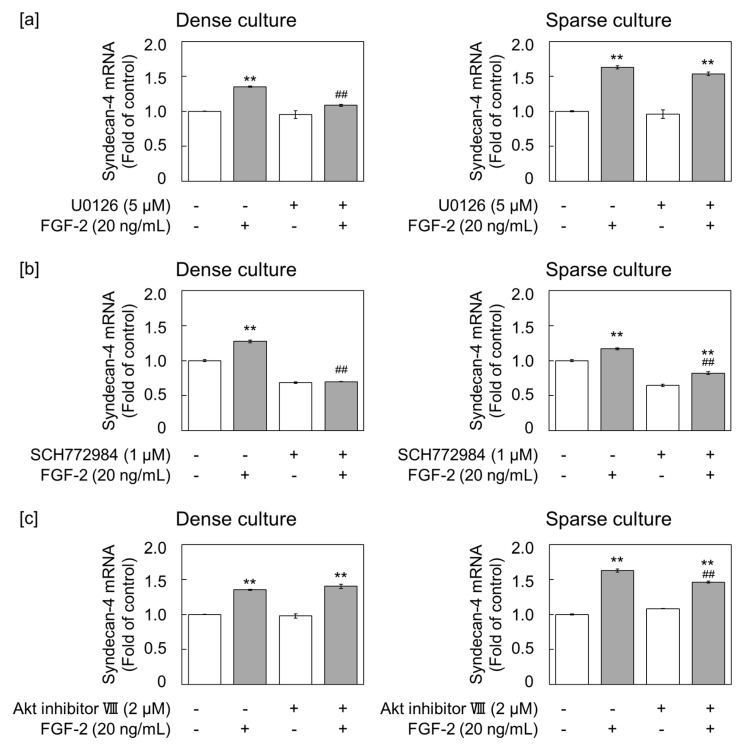
Effects of inhibition of MEK1/2, ERK1/2, and Akt on the expression of syndecan-4 mRNA in dense and sparse cultures of vascular endothelial cells. Vascular endothelial cells in dense and sparse cultures were pretreated with the (**a**) 5 µM MEK1/2 inhibitor U0126, (**b**) 1 µM ERK1/2 inhibitor SCH772984, or (**c**) 2 µM Akt inhibitor (Akt inhibitor VIII) at 37 °C for 3 h, followed by stimulation with or without 20 ng/mL FGF-2 for 6 h. The syndecan-4 mRNA level was quantified using qRT-PCR. Values represent the mean ± S.E. of four technical replicates. ** *p* < 0.01, significantly different from the corresponding treatment without FGF-2; ^##^ *p* < 0.01, significantly different from the corresponding inhibitor treatment.

**Figure 5:**
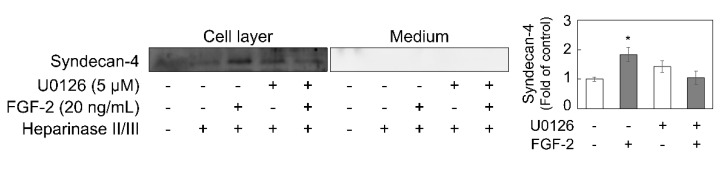
Effects of MEK1/2 inhibitor U0126 on the expression of syndecan-4 core protein expression in the dense culture of vascular endothelial cells. The dense culture of vascular endothelial cells was pretreated with 5 µM MEK1/2 inhibitor U0126 at 37 °C for 3 h and then stimulated with 20 ng/mL FGF-2 for 6 h, and assessed for the syndecan-4 core protein level by western blotting. The bar graph shows the intensity of syndecan-4 in the cell layer in the group treated with heparinase II/III. The values in the bar graphs indicate the means ± S.E. of three samples of the experiments. * Significantly different from the control, *p* < 0.05.

To examine the involvement of ERK1/2 and Akt in the regulation of syndecan-4 expression by FGF-2, dense and sparse cultures of vascular endothelial cells were pretreated with MEK1/2 (known as ERK1/2 kinase) inhibitor U0126, ERK1/2 inhibitor SCH772984, or Akt inhibitor VIII for 3 h, and then stimulated with 20 ng/mL FGF-2 for 6 h. U0126 was found to suppress FGF-2-induced syndecan-4 mRNA expression in the dense cell culture, with no significant effect observed in the sparse cell culture (Figure 4a). The constitutive expression of syndecan-4 mRNA was reduced by SCH772984 alone in both dense and sparse cultures; however, FGF-2-induced syndecan-4 upregulation was only completely suppressed by this inhibitor in the dense culture (Figure 4b). These data suggest that FGF-2 induces syndecan-4 mRNA via the MEK1/2-ERK1/2 pathway. In contrast, the inhibition of Akt only partly suppressed FGF-2-induced syndecan-4 mRNA expression in the low density culture, without conferring any effect at a high cell density (Figure 4c). Corroborating this, U0126 suppressed FGF-2-mediated syndecan-4 core protein induction in the cell layer from the dense culture (Figure 5).

## 3. Discussion

In this study, the cell density-dependent regulation of proteoglycans by FGF-2 was investigated using a culture system of bovine aortic endothelial cells. The results indicate that FGF-2 induced endothelial syndecan-4 transcript expression, regardless of the cell density, in a dose-dependent manner; however, the mechanism of syndecan-4 core protein expression was cell-density-dependent. Specifically, the expression of syndecan-4 was upregulated by FGF-2 via the MEK1/2-ERK1/2 pathway at a high cell density, whereas only the transcriptional induction of syndecan-4 occurred via the Akt pathway at a low cell density. It was found, for the first time, that syndecan-4 is induced by FGF-2 in vascular endothelial cells, and the cell-density-dependent regulation of its expression via distinct signaling pathways was revealed. We previously reported several growth factors that modify the expression and characteristics of proteoglycans in vascular endothelial cells [18,19,22]. For example, vascular endothelial growth factor (VEGF) and TGF-β_1_ enhance perlecan core protein synthesis when the cell density is high [18,22]. In addition, we recently reported that endothelial syndecan-4 induction depends on the activation of p38 MAPK, but not Smad2/3, by an organocopper complex [23,24]. However, the present data shows that FGF-2 upregulates syndecan-4 expression without the activation of p38 MAPK. Therefore, it is indicated that vascular endothelial cells use different signaling pathways to modulate the expression of syndecan-4, depending on the external cues, such as growth factors and chemical compounds.

Heparan sulfate proteoglycans synthesized by vascular endothelial cells have two important physiological functions; a heparin-like anticoagulant activity to prevent intravascular coagulation and a co-receptor activity for FGF-2 to repair damaged endothelium [7,25]. Syndecan-4, a focal adhesion molecule that plays an important role in the alignment of endothelial cells along the blood stream [26,27], was originally isolated from rat footpad endothelial cells as a molecule with the ability to bind antithrombin III [12]. Heparan sulfate chains are responsible for the interaction of the syndecan-4 molecule with antithrombin III and thrombin. The inhibition of thrombin activity by antithrombin III is largely elevated by forming a ternary complex of thrombin, antithrombin III, and heparan sulfate chains [28,29]. Moreover, studies on heparan sulfate proteoglycan expression have reported that FGF-2 is secreted in close proximity to the cell surface by an unconventional pathway in dense cultures and promotes the anticoagulant activity of vascular endothelial cells near the damaged endothelium, thereby enabling turnover of the endothelium [30,31]. Conversely, per cell, the amount of FGF-2 released, FGF-2 binding capacity, and FGF-2 stimulation of cell growth is higher in vascular endothelial cells at a lower cell density [32,33]. Moreover, syndecan-4 has been shown to support FGF-2-induced proliferation, as well as the tube formation of vascular endothelial cells [34]. Our current findings, together with the aforementioned observations, indicate that the induction of syndecan-4 by FGF-2 in dense cultures of vascular endothelial cells may help in effectuating the repair of a damaged vascular endothelial cell layer. In addition, FGF-2-induced syndecan-4 may be involved in angiogenesis [35]; it has been reported that immune cells, e.g., T cells, positively [36] or negatively [37] regulate vascular regeneration by modulating the signaling from angiogenic factors, such as FGF-2, IL-10, VEGF, and TGF-β, that regulate the proliferation of vascular endothelial cells in vivo.

The present data showed that FGF-2 upregulates the expression of syndecan-4 mRNA and also leads to the activation of ERK1/2 and Akt in dense and sparse cultures of vascular endothelial cells, respectively. It was further shown that the syndecan-4 core protein is only induced by FGF-2 via the MEK1/2-ERK1/2 pathway in a dense culture of vascular endothelial cells. As can be seen in Figure 3, vascular endothelial cells express more ERK1 (p44) than ERK2 (p42) in both dense and sparse cultures. When the cell density is high, the phosphorylated ERK2 detected is slightly stronger than that of ERK1, and the phosphorylation of ERK2 is enhanced by FGF-2 treatment. In contrast to that, in the dense culture, phosphorylated ERK2 was nearly undetectable in the sparse culture of vascular endothelial cells, irrespective of FGF-2 treatment. It would be interesting to assess whether ERK1 and ERK2 phosphorylation differentially affect the induction of the syndecan-4 core protein by FGF-2, as some evidence highlights the specific roles of ERK1 and ERK2 in vascular endothelial cells at different cell densities [38]. In the present study, syndecan-4 core protein induction by FGF-2 was only observed when the cell density was high. Since sparse cultures lacked intracellular contacts, it is suggested that cell–cell contact is required for syndecan-4 core protein induction by FGF-2 in vascular endothelial cells. It has been reported that the synthesis of biglycan, a small dermatan sulfate proteoglycan, is modulated by FGF-2 in migrating endothelial cells [39]. This suggests that there is a relationship between the regulation of proteoglycan synthesis and endothelial cell behavior. Moreover, it is known that syndecan-4 contributes to angiogenesis and FGF-2-induced chemotactic migration [40,41]. Therefore, the induction of syndecan-4 by FGF-2 may be involved in the regulation of vascular endothelial cell behavior, such as angiogenesis.

Our study shows, for the first time, that the proteoglycan synthesis in vascular endothelial cells is modulated by FGF-2 via distinct signaling pathways, depending on the cell density. FGF-2 upregulates the synthesis of syndecan-4 in vascular endothelial cells via the MEK1/2-ERK1/2 pathway when the cell density is high, whereas, at a low cell density, only the transcriptional induction of syndecan-4 occurs via the Akt pathway. The present study revealed that not only TGF-β, which modulates perlecan and biglycan synthesis, but also FGF-2, modulate syndecan-4 expression in a cell density-dependent manner. This regulation by FGF-2 may be a part of the wide array of mechanisms underlying the proteoglycan-mediated regulation of endothelial cell functions by FGF-2.

## 4. Materials and Methods

### 4.1. Materials

Bovine aortic endothelial cells, identified by acetylated low density lipoprotein uptake and VE-cadherin/cluster of differentiation 31 (CD31) mRNA expression, were purchased from Cell Applications (San Diego, CA, USA). Dulbecco’s modified Eagle’s medium (DMEM) and Ca^2+^- and Mg^2+^-free phosphate-buffered saline (PBS) were obtained from Nissui Pharmaceutical (Tokyo, Japan). Fetal bovine serum (FBS) was obtained from Biosera (Kansas City, MO, USA). The BCA protein assay kit and high-capacity cDNA reverse transcription kit were purchased from Thermo Fisher Scientific (Waltham, MA, USA). Recombinant human FGF-2 (067-04031) and the mouse monoclonal antibody against β-actin were obtained from Wako Pure Chemical Industries (Osaka, Japan). Chemically synthetic U0126, SCH772984, and Akt inhibitor VIII were purchased from Cayman Chemical (Ann Arbor, MI, USA). Heparinase II (derived from *Flavobacterium heparinum*) and heparinase III (EC 4.2.2.8, derived from *F. heparinum*) were acquired from IBEX Technologies (Montreal, QC, Canada). Diethylaminoethyl (DEAE)-Sephacel was obtained from Sigma-Aldrich (St Louis, MO, USA). Anti-ERK1/2 (#9102), anti-phospho-ERK1/2 (#9101), anti-JNK (#9252), anti-phospho-JNK (#9255), anti-p38 MAPK (#9212), anti-phospho-p38 MAPK (#9211), anti-Akt (#4060), anti-phospho-Akt (#4691), horseradish peroxidase (HRP)-conjugated anti-rabbit (#7074), and HRP-conjugated anti-mouse (#7076) IgG antibodies were obtained from Cell Signaling Technology (Beverly, MA, USA). Goat polyclonal antibody against syndecan-4 (N-19) was obtained from Santa Cruz Biotechnology (Santa Cruz, CA, USA). Horseradish peroxidase-conjugated anti-goat IgG antibody (ab6885) was obtained from Abcam (Bristol, UK). Immobilon-P polyvinyl difluoride (PVDF) membrane was purchased from Millipore (Billerica, MA, USA). QIAzol lysis reagent was obtained from QIAgen (Hilden, Germany). GeneAce SYBR qPCR mix α was acquired from Nippon Gene (Tokyo, Japan). Other reagents, of the highest grade available, were purchased from Nacalai Tesque (Kyoto, Japan).

### 4.2. Cell Culture and Treatments

Vascular endothelial cells were cultured in DMEM containing 10% FBS and maintained in a humidified atmosphere with 5% CO_2_ at 37 °C until confluent. The cells were then transferred into 35 mm dishes and cultured until confluent (“dense culture”) or into 100 mm dishes at 1 × 10^4^ cells/cm^2^ and cultured for 24 h (“sparse culture”). The cell density before treatment with FGF-2 or inhibitors was 1.50 × 10^5^ cells/cm^2^ (dense culture) and 1.25 × 10^4^ cells/cm^2^ (sparse culture). Sparse cultures lacked intracellular contacts. For western blot analysis, both dense and sparse cultures were prepared in 100 mm dishes. The medium was discarded and the cells were washed twice with serum-free DMEM; the cells were then treated with FGF-2 (10, 20, 50, or 100 ng/mL) for 0.5, 1, 2, 4, 6, 8, 12, 16, 20, or 24 h, in the presence or absence of MEK1/2 inhibitor U0126 (5 µM), ERK1/2 inhibitor SCH772984 (1 µM), or Akt inhibitor VIII (2 µM). Experiments were performed after confirming that the inhibitors suppressed the activation of corresponding signal molecules in both dense and sparse cultures, depending on the concentration at the pretreatment time tested in this study. The detailed conditions are provided in figure legends. After treatment, the following experiments were performed. 

### 4.3. Quantitative Reverse Transcription Polymerase Chain Reaction (qRT-PCR)

Extraction of the total RNA from vascular endothelial cells was performed as previously described [42]. Briefly, vascular endothelial cells were washed twice with PBS and lysed with QIAzol lysis reagent. The lysate was mixed with a one-fourth volume of chloroform and centrifuged to obtain an aqueous supernatant layer. The supernatant was harvested and 70% ethanol was added at a concentration of 52.5%. The suspension was centrifuged at 20,000× *g*, and the precipitate was obtained. The RNA precipitate was washed with 70% ethanol and centrifuged at 20,000× *g*; the precipitate was collected and dried. The total RNA was used for the synthesis of complementary DNA with a high-capacity cDNA reverse transcription kit. qRT-PCR was performed using GeneAce SYBR qPCR mix α with 1 ng/µL cDNA and 0.1 µM primers (Table 1) in a StepOnePlus^TM^ real-time PCR system (Thermo Fisher Scientific). Perlecan, syndecan-1, syndecan-2, syndecan-3, syndecan-4, biglycan, and glyceraldehyde 3-phosphate dehydrogenase (GAPDH) transcript levels were analyzed quantitatively, using the relative standard curve method. The fold change in intensity of the target gene was normalized to that of GAPDH. qRT-PCR was performed in four technical replicates and the reproducibility was confirmed by using samples obtained from independent experiments, at least twice.

### 4.4. Proteoglycan Core Protein Extraction and Western Blot Analysis

Proteoglycans accumulated in the cell layer and the conditioned medium was extracted and concentrated as previously described [42]. To degrade heparan sulfate chains, they were dissolved with 100 mM Tris-HCl buffer (pH 7.0) containing 10 mM calcium acetate and 18 mM sodium acetate in the presence of 0.02 IU/mL heparinase II/III for 3 h at 37 °C. The core proteins were dissolved in sodium dodecyl sulfate (SDS) sample buffer ((50 mM Tris-HCl buffer solution containing 2% SDS and 10% glycerol (pH 6.8)) and incubated at 95 °C for 3 min. The proteins were then separated by SDS-polyacrylamide gel electrophoresis on a 4–16% polyacrylamide slab gel, as previously described [43]. Syndecan-4 core protein was detected at a molecular mass of approximately 40,000 as an SDS-resistant dimer [44]. Western blot analysis was performed using samples obtained from independent experiments to confirm reproducibility. The values in the bar graphs indicate the means ± S.E. of three samples of the experiments. 

### 4.5. Statistical Analysis

Data were analyzed for statistical significance using a Student’s *t*-test (Figure 2 and Figure 3) or Tukey’method after ANOVA analysis (Figure 1, Figure 4, and Figure 5). *p* < 0.05 was considered to be statistically significant.

## Figures and Tables

**Table 1:** Bovine gene-specific primers for qRT-PCR.

Gene	Forward Primer (5′–3′)	Reverse Primer (5′–3′)
Perlecan	GCTGAGGGCGTACGATGG	TGCCCAGGCGTCGGAACT
Syndecan-1	CAGTCAGGAGACAGCATCAG	CCGACAGACATTCCATACC
Syndecan-2	CCAGATGAAGAGGACACAAACG	CCAATAACTCCGCCAGCAA
Syndecan-3	CAAGCAGGCGAGCGTC	GGTGGCAGAGATGAAGTGG
Syndecan-4	TTGCCGTCTTCCTCGTGC	AGGCGTAGAACTCATTGGTGG
Biglycan	GCTGCCACTGCCATCTGAG	CGAGGACCAAGGCGTAG
GAPDH	AACACCCTCAAGATTGTCAGCAA	ACAGTCTTCTGGGTGGCAGTGA

## Supplementary Materials

Supplementary materials can be found at https://www.mdpi.com/1422-0067/21/10/3698/s1. Figure S1: Dose-dependent effects of FGF-2 on perlecan core protein expression in dense 1 and sparse cultures of vascular endothelial cells. Dense vascular endothelial cells were treated with 2 FGF-2 at 10, 20, 50, 100 ng/mL at 37 °C for 8 h.

## Abbreviations

DEAE	Diethylaminoethyl
DMEM	Dulbecco’s modified Eagle’s medium
ERK	Extracellular signal-regulated kinase
FBS	Fetal bovine serum
FGF-2	Fibroblast growth factor-2
GAPDH	Glyceraldehyde 3-phosphate dehydrogenase
HRP	Horseradish peroxidase
JNK	c-Jun N-terminal kinase
MAPK	Mitogen-activated protein kinase
PVDF	Polyvinyl difluoride
qRT-PCR	Quantitative reverse transcription polymerase chain reaction
SDS	Sodium dodecyl sulfate
TGF-β	Transforming growth factor-β
VEGF	Vascular endothelial growth factor
