# Supplementary material for: Cell Density-Dependent Fibroblast Growth Factor-2 Signaling Regulates Syndecan-4 Expression in Cultured Vascular Endothelial Cells
Source: Int J Mol Sci. 2020 May 24;21(10):3698. doi: 10.3390/ijms21103698 (PMC7279341; doi:10.3390/ijms21103698)
Supplement: Supplementary file 1 [file ijms-21-03698-s001.pdf]

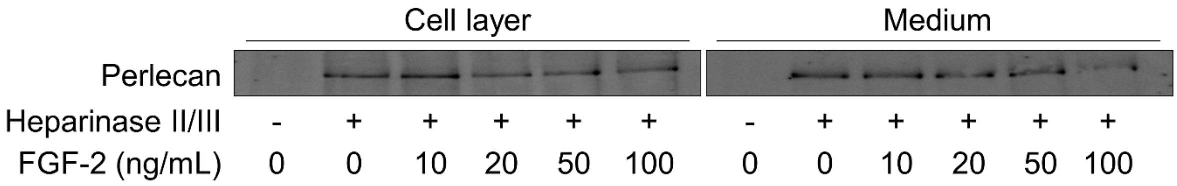

Supplemental figure 1. Dose-dependent effects of FGF-2 on perlecan core protein expression in dense and sparse cultures of vascular endothelial cells. Dense vascular endothelial cells were treated with FGF-2 at 10, 20, 50, 100 ng/mL at 37 °C for 8 h.
